# Advances in the Study of Necroptosis in Vascular Dementia: Focus on Blood–Brain Barrier and Neuroinflammation

**DOI:** 10.1111/cns.70224

**Published:** 2025-02-06

**Authors:** Yuemin Qiu, Lin Cheng, Yinyi Xiong, Ziying Liu, Chunxiao Shen, Liangliang Wang, Yujia Lu, Shufei Wei, Lushun Zhang, Seung Bum Yang, Xiaorong Zhang

**Affiliations:** ^1^ Department of Pathology Affiliated Hospital of Jiujiang University Jiujiang Jiangxi China; ^2^ Department of Pathology Jiujiang Clinical Precision Medicine Research Center Jiujiang Jiangxi China; ^3^ Department of Neurology Affiliated Hospital of Jiujiang University Jiujiang Jiangxi China; ^4^ Department of Rehabilitation Affiliated Hospital of Jiujiang University Jiujiang Jiangxi China; ^5^ Department of Medical Non‐Commissioned Officer Wonkwang Health Science University Iksan Republic of Korea

**Keywords:** blood–brain barrier, necroptosis, neuroinflammation, pathophysiology, vascular dementia

## Abstract

**Background:**

Vascular dementia (VaD) includes a group of brain disorders that are characterized by cerebrovascular pathology.Neuroinflammation, disruption of the blood–brain barrier (BBB) permeability, white matter lesions, and neuronal loss are all significant pathological manifestations of VaD and play a key role in disease progression. Necroptosis, also known asprogrammed necrosis, is a mode of programmed cell death distinct from apoptosis and is closely associated with ischemic injury and neurodegenerative diseases. Recent studies have shown that necroptosis in VaD exacerbates BBB destruction, activates neuroinflammation, promotes neuronal loss, and severely affects VaD prognosis.

**Results and Conclusions:**

In this review, we outline the significant roles of necroptosis and its molecular mechanisms in the pathological process of VaD, with a particular focus on the role of necroptosis in modulating neuroinflammation and exacerbating the disruption of BBB permeability in VaD, and elaborate on the molecular regulatory mechanisms and the centrally involved cells of necroptosis mediated by tumor necrosis factor‐α in neuroinflammation in VaD. We also analyze the possibility and specific strategy that targeting necroptosis would help inhibit neuroinflammation and BBB destruction in VaD. With a focus on necroptosis, this study delved into its impact on the pathological changes and prognosis of VaD to provide new treatment ideas.

## Graphical abstract

Neuroinflammation and blood–brain barrier disruption play key roles in the progression of vascular dementia, and necroptosis is closely associated with vascular dementia. This review guides the prevention and treatment of vascular dementia by discussing the tumor necrosis factor‐α signaling pathway, destruction of endothelial cells, glial cells, and oxidative stress and mitochondrial dysfunction in the presence of necroptosis. Inhibition of necroptosis may be useful in the treatment of vascular dementia to unfold a new world.

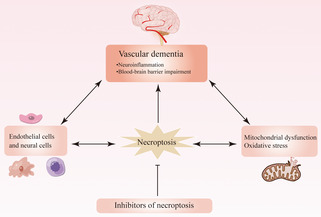

## 1 Introduction

Vascular dementia (VaD) results in cognitive impairment and memory loss and is the second‐most common type of dementia worldwide after Alzheimer's disease (AD) [1, 2, 3, 4]. With the aging of the population, the incidence of VaD is increasing, bringing a heavy economic burden to families and society, and has become a public health problem that seriously threatens human health, but its underlying pathogenesis has not yet been fully elucidated. Given the lack of identifiable therapeutic targets, there is an urgent need to identify effective treatment modalities [5, 6]. Therefore, in‐depth exploration of the pathogenesis of VaD to identify therapeutic targets is critical. Numerous clinical studies have found that pro‐inflammatory mediators, such as cytokines, chemokines, and interleukins (IL) are significantly elevated in the peripheral blood of patients with VaD. At the same time, reactive microglia and astrocytes around the damaged white matter are significantly activated, indicating that neuroinflammation is a meaningful pathological change of VaD [7, 8]. Additionally, inhibition of glial cell activation and abatement of neuroinflammation are effective in improving cognitive impairment in animal models of VaD [9, 10, 11]. Therefore, suppressing neuroinflammation is vital to improving cognitive impairment in VaD. The destruction of the blood–brain barrier (BBB) is a significant cause for aggravating neuroinflammation, leading to neuronal loss [12, 13]. BBB permeability in the brain also increased with increased white matter lesions in patients with VaD [14]. Thus, neuroinflammation and BBB disruption play a key role in the progression of VaD.

Necroptosis is a form of regulated necrotic cell death mediated by receptor‐interacting protein kinase 1 (RIPK1) kinase activity, RIPK3, and mixed‐lineage kinase domain‐like pseudokinase (MLKL) [15, 16, 17]. An increasing number of studies have shown that necroptosis is closely related to age‐related neurodegenerative diseases and acute neuronal injury [18, 19, 20, 21, 22]. Necroptosis mediates endothelial cell (EC) death and neuroinflammation and participates in BBB decomposition after cerebral ischemic/reperfusion injury [23, 24, 25, 26]. It has been found that inhibition of RIPK1, RIPK3, and MLKL in necroptosis can rescue neuronal cell loss in xenografted human neurons [21]. Furthermore, the involvement of necroptosis in regulating neuroinflammation and BBB destruction, which mediates cortical neuronal death, has been demonstrated in both cellular and animal models of ischemic brain injury and is closely associated with cognitive deficits in VaD; the possible mechanism is closely related to the RIPK3‐mediated inflammatory signaling pathway inducing apoptotic neuronal death in VaD [27, 28, 29, 30, 31, 32].

To sum up, the pathogenesis of VaD is very complex, and there is no effective treatment at present. Necroptosis is closely related to VaD, but its exact molecular mechanism remains unclear. This review discusses the molecular mechanism between necroptosis and neuroinflammation and between VaD and the destruction of BBB permeability to guide the prevention and treatment of VaD. Elucidating this mechanism can provide a new perspective for developing the prevention and treatment of VaD.

## 2 Overview of VaD

Currently, VaD has various clinical diagnostic criteria, which, despite their different characteristics, cover two core elements: (1) to confirm the existence of dementia and (2) to identify vascular disease as the primary pathological cause of dementia [33, 34, 35, 36, 37, 38]. Although the underlying mechanism leading to the development of VaD has not been fully elucidated, many studies have clearly shown that the pathogenesis of VaD is closely related to many vascular‐related risk factors, including diabetes, hypertension, and hyperlipidemia, which can lead to macrovascular and microvascular lesions, which promote vascular changes in the cerebral cortex and subcortex, including white matter hyperintensity, lacunar infarction, and microhemorrhage, resulting in VaD [39, 40, 41, 42, 43, 44]. Ischemia and hypoxia‐induced by long‐term chronic cerebral hypoperfusion (CCH) can trigger cascade reactions of molecular and cellular injury, over‐activate neuroinflammation, lead to pathological changes, such as BBB damage, and promote the occurrence and progression of VaD [45, 46].

**FIGURE 1:**
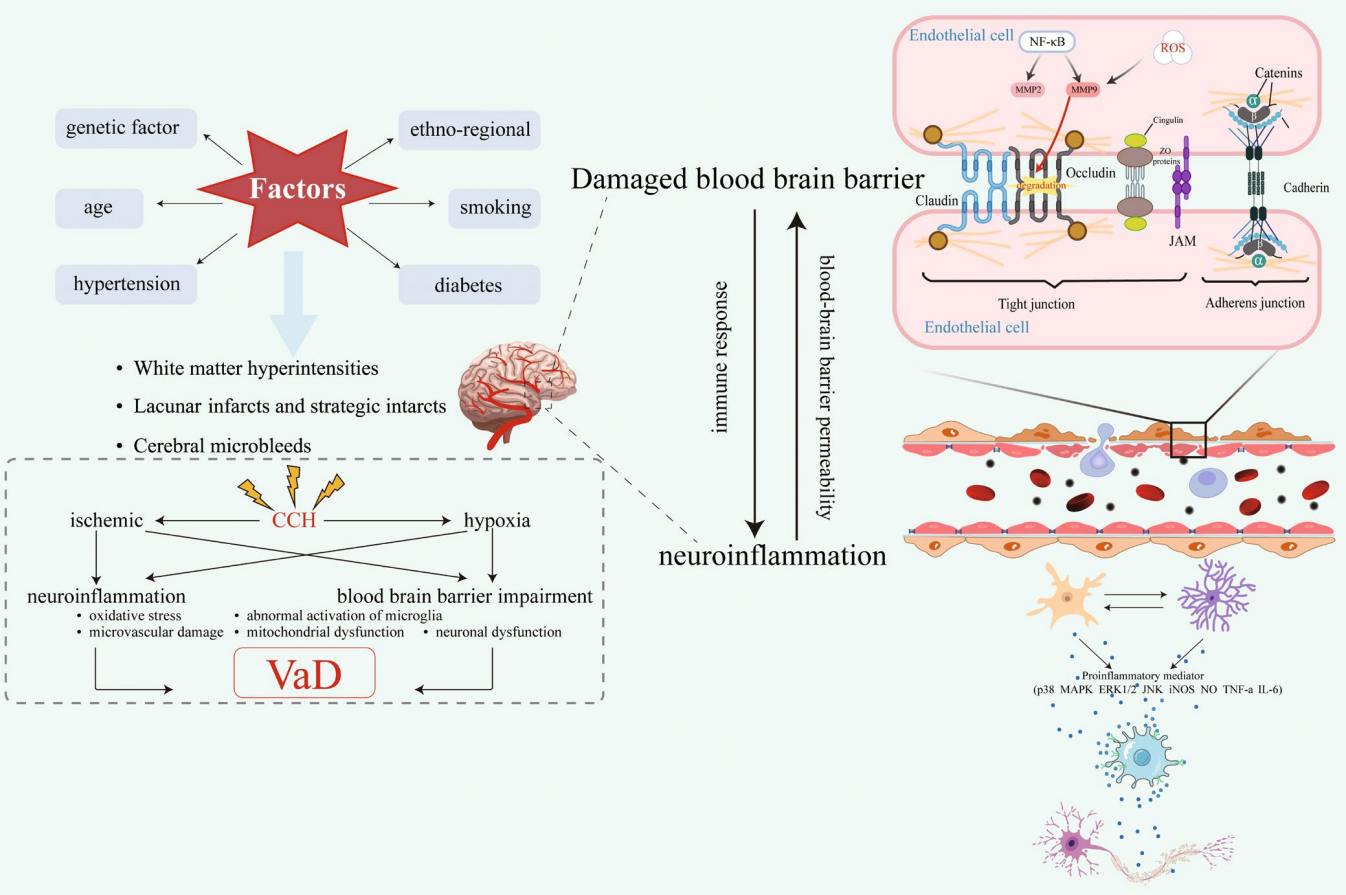
Overview of VaD and the impact between neuroinflammation and the blood–brain barrier. Risk factors leading to cortical and subcortical vascular changes and long‐term CCH promote VaD development. Activated microglia and reactive astrocytes interact with each other and release large quantities of pro‐inflammatory mediators that, in turn, damage neurons and cause neuronal death. The BBB consists of ECs, pericytes, astrocyte endfeet, and basement membranes. The junctional complexes between ECs include tight junctions (TJs) and adherens junctions (AJs). TJs structures include transmembrane proteins (occludin, claudins, and junctional adhesion molecules); actin filaments; and cytoplasmic scaffolding proteins (zonula occluden [ZO]). Junctional adhesion molecules (JAMs) are part of TJs and contribute to the adhesion of EC membranes. AJs play a role in maintaining the structural stability of TJs.

Neuroinflammation and BBB destruction are the basic pathological manifestations of VaD and influence each other. Neuroinflammation can increase the permeability of BBB. Conversely, increased BBB permeability can also lead to persistent immune response and chronic neuroinflammatory state, thereby aggravating the vicious circle of the VaD pathological process [47, 48]. Therefore, the focus of this review will be on neuroinflammation and BBB disruption in VaD states (Figure 1).

### 2.1 Neuroinflammation in VaD States

Neuroinflammation is thought to be the main pathogenic factor of VaD [49, 50]. Neuroinflammation in the central nervous system (CNS) is primarily an immune cascade mediated by glial cells due to CNS injury, infection, toxicity, or autoimmunity, eventually leading to neuronal or axonal degeneration or death [51, 52]. Immune cells involved in this process include microglia and astrocytes, but oligodendrocytes are closely related to neuroinflammation. Myelin‐associated antigen excitations released by oligodendrocytes can activate peripheral T cells, leading to an autoimmune response. After ischemic stroke, oligodendrocytes derived from oligodendrocyte precursor cells participate in neuroinflammation and contribute to the recovery of nerve function [53]. In addition, neuroinflammation can also deplete oligodendrocytes, the demyelination and remyelination of oligodendrocytes [54]. Neuroinflammation caused by astrocytic and microglial activation was found to be involved in the hippocampal damage process in VaD rats in a study by Sun et al. [55]. Previous studies have demonstrated that activated glial cells induce neuroinflammation via activation of pro‐inflammatory mediators, such as mitogen‐activated protein kinase (MAPK) p38, extracellular signal‐regulated kinase 1/2, c‐Jun N‐terminal kinase, inducible nitric oxide synthase (iNOS), nitric oxide (NO), tumor necrosis factor‐α (TNF‐α), and IL‐6 [56, 57]. Multiple studies have shown that abnormal activation of microglia in VaD is also an important manifestation of neuroinflammation, and activated microglia can lead to neuronal cell death, tissue matrix degradation, BBB dysfunction, and myelin damage [4, 58, 59, 60, 61]. The activation of microglia and reduction of neuroinflammation promote neurogenesis and ameliorate cognitive dysfunction in the VaD mouse model [10]. The above evidence suggests that neuroinflammation is an important pathological manifestation of VaD, and inhibition of neuroinflammation can significantly improve cognitive impairment in VaD.

### 2.2 BBB Damage in VaD States

The BBB comprises ECs, pericytes, astrocyte endfeet, and basement membranes, which together in their closely related roles with neurons form the neurovascular unit (NVU) [62, 63, 64, 65]. Loss of ECs integrity, pericyte degeneration, astrocyte internal membrane swelling, retraction from the vessel wall, loss of tight junctions (TJs) proteins, and cerebral capillary leakage can disrupt the integrity of the BBB, which subsequently leads to CCH [66, 67, 68]. Moreover, prolonged CCH also leads to further reductions in ZO‐1, occludin, and claudin‐5 in TJs, leading to BBB dysfunction and neuroinflammatory responses [69]. The results of animal studies showed that the expression of claudin‐5 protein, which maintains the structure and function of the BBB, was significantly reduced in the hippocampus of the VaD mouse model, indicating that the BBB structure was disrupted [70]. In addition, matrix metalloproteinases (MMPs), such as MMP‐9 and MMP‐2, cleave the extracellular matrix and degrade the TJs of the nuclear basal lamina between ECs, which are essential for the maintenance of the BBB [71]. More convincing evidence shows a significant increase of MMP‐2 in the brain and serum of CCH mice and an increase of MMP‐9 in the serum of VaD patients [72]. Taken together, these results suggest that increased BBB permeability is an essential pathological manifestation of VaD and that its mechanism of action is related to MMP‐2 and MMP‐9. However, their mechanisms of action in CCH and VaD have not been fully elucidated.

BBB damage is an early pathological change in cognitive impairment, and BBB damage is considered the leading cause, rather than the result, of aging‐related neurodegenerative diseases [73, 74]. Recent studies show that BBB permeability and WM damage are important pathways by which vascular load adversely affects cognitive functioning [75]. The researchers observed loss of capillary pericytes and BBB in the WM of patients with VaD [76]. Furthermore, during disease progression, increased BBB permeability in VaD is accompanied by neuronal loss and WM degeneration [77]. Therefore, BBB destruction is an early change, which has important prognostic value in VaD and provides a critical window of time for treatment.

## 3 Necroptosis Promotes Neuroinflammation and Aggravates VaD

Necroptosis is mainly initiated by members of the TNF receptor (TNFR) and Toll‐like receptor (TLR) families, interferon, intracellular RNA and DNA sensors, and other mediators [78, 79, 80]. TNF‐α stimulates TNFR1, inducing its activation leading to necroptosis or RIPK1‐dependent apoptosis [79, 81, 82]. It is worth mentioning that activation of RIPK1, a pivotal kinase in necroptosis, is one of the main mechanisms of injury in ischemic brain damage [32]. It has been shown that inhibition of RIPK1 activation and TNF‐α‐induced necroptosis can effectively reduce neuroinflammation and ameliorate brain injury [83, 84]. A variety of inflammatory cytokines, including iNOS, TNF‐α, and IL‐6, can promote neuronal cell death and BBB destruction [85, 86]. High expression of iNOS and excessive production of NO can also induce neuroinflammation and lead to necroptosis of neurons [87]. In the PND21 mouse model, arsenic‐activated microglia via the p38 MAPK signaling pathway led to the overproduction and release of TNF‐α. Microglia‐derived TNF‐α then recruits RIPK1 and RIPK3, activated MLKL, and ultimately induces neuronal necroptosis [88]. In this review, we focus on the effect of necroptosis activated by TNF‐α and TNFR1 on neuroinflammation in VaD and its mechanism of action.

### 3.1 TNF‐α Modulates Necroptosis to Exacerbate Neuroinflammation in VaD

**FIGURE 2:**
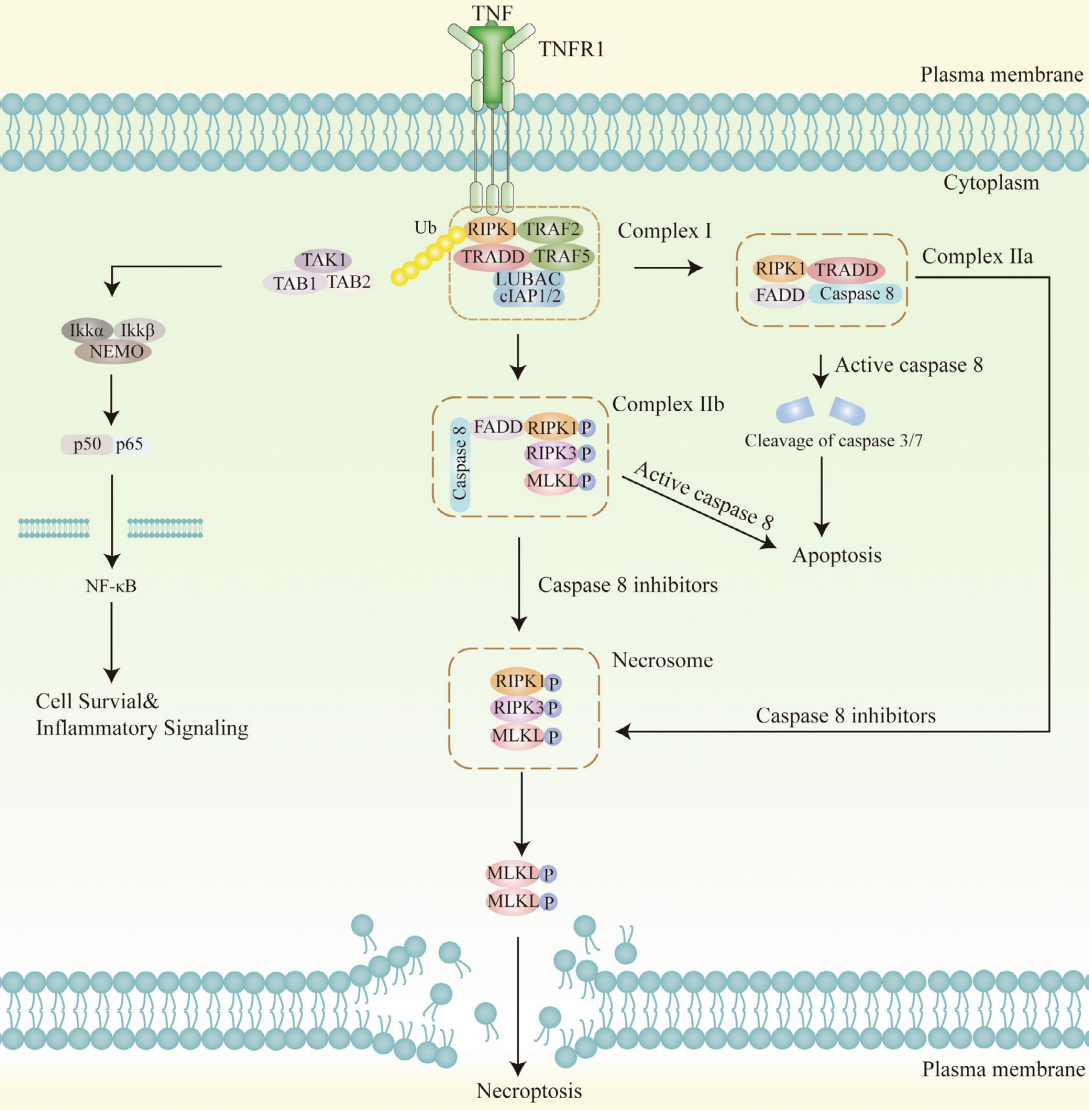
TNF‐α/TNFR1 mediates the major signaling pathway of necroptosis. The binding of TNFR1 to TNF trimer leads to the formation of complex I at the cytoplasmic membrane consisting of TRADD, TRAF2, RIPK1, CYLD, and cIAP1/cIAP2. cIAP1/cIAP2 ubiquitinates RIPK1, activates the IKK complex, leading to the activation of NF‐κB and the activation of survival pathways. In the absence of cIAP1/cIAP2, RIPK1, TRADD, FADD, and caspase‐8 form complex IIa. The high local concentration of pro‐caspase 8 induces caspase 8 activation through autocleavage and trans‐cleavage from other active caspases. Caspase 8 then cleaves caspase 3 and caspase 7 and then induces apoptosis. When caspase‐8 activity is inhibited, complex IIb becomes the dominant signaling pathway. RIPK1, RIPK3, and MLKL form necrosomes and accelerate the polymerization of MLKL to form oligomers that are transported to the plasma membrane. MLKL binds to phosphatidylinositol phosphates on the inner membrane, disrupting the integrity of the cell membrane and leading to the release of intracellular contents. This process leads to cell swelling, rupture, and ultimately cell necroptosis.

TNF‐α is one of the most important pro‐inflammatory cytokines secreted by various cells, including macrophages and microglia, and regulates necroptosis through the paracrine system [89]. When TNF‐α binds to TNFR1 on the membrane surface, it prompts the rapid formation of TNFR1 complex I. This complex I mainly comprises TNFR‐associated death domain protein (TRADD), RIPK1, TNFR‐associated factor 2 (TRAF2), and cellular inhibitor of apoptosis 1/2 (cIAP1/2) as well as linear ubiquitin chain assembly complex (LUBAC) [52, 90]. Ubiquitinated RIPK1 activates the nuclear factor κB (NF‐κB) pathway to initiate cell survival pathways and inflammatory responses. When the NF‐κB signaling pathway is inhibited, TRADD in complex I recruits Fas‐associated death domain (FADD) and caspase‐8 to form complex IIa, and when RIPK1 is deubiquitylated, necroptosis‐related complex IIb is formed by RIPK1, FADD, and caspase‐8 formation [91]. When caspase‐8 is inhibited, microsomes can be formed, comprising mainly of RIPK1 and RIPK3 heterodimerization [92]. There are three main events downstream of TNF‐α‐mediated necroptosis: phosphorylation of MLKL, assembly of MLKL‐containing macromolecular complexes, and disruption of cell membranes [93, 94, 95] (Figure 2).

Following phosphorylation and oligomerization of MLKL in RIPK3‐phosphorylated microsomes, the N‐terminus of MLKL binds directly to phosphatidylinositol phosphates, enabling translocation to the membrane compartment, which leads to membrane rupture and release of cellular contents [96]. In the study by Xu et al. [82] activated neuronal necroptosis is dependent on upstream TNF‐α/TNFR1 signaling in both neuronal cell cultures and AD mouse models. It has been demonstrated that ischemic brain injury triggers necroptosis by activating RIPK associated with TNF‐α/death receptor, which promotes neuronal cell death [97]. Elevated TNF levels were found in VaD patients' cerebrospinal fluid, suggesting the important role of TNF‐α‐mediated necroptosis in VaD [98]. By contrast, necroptosis is characterized by plasma membrane rupture, cell swelling, and loss of cell and organelle integrity [99]. Plasma membrane rupture leads to the efflux of cytokines, chemokines, and potassium and the release of damage‐associated molecular patterns (DAMPs), triggering activation of the immune system, and neuroinflammation so that necroptosis is highly pro‐inflammatory [100, 101, 102, 103]. This is supported by a study in which necroptosis and its accompanying inflammatory response could lead to acute injury following ischemic stroke [104]. Necroptosis cells also secrete extracellular vesicles that may be able to modulate the inflammatory response of surrounding cells and are emerging as an important mediator of pro‐inflammatory signaling during cell death [105, 106, 107]. In summary, TNF‐α‐mediated necroptosis is closely associated with neuroinflammation in VaD, but its specific molecular mechanism remains to be thoroughly investigated.

### 3.2 The Effect of Necroptosis of Microglia and Astrocytes on Neuroinflammation

Microglia are immune cells in the brain that maintain the immune defense of the nervous system [108, 109, 110]. Microglia are classified into two subtypes: classically activated (M1) cells, the phenotype more likely to be associated with neuroinflammation in neurodegenerative diseases, and alternatively activated (M2) cells, the phenotype promoting inflammatory regression and tissue repair [111, 112, 113]. In patients with VaD, a large accumulation of phagocytic activated microglia is seen in the perivascular periphery of the stroke focus [61]. In the CNS, neuroinflammation is characterized by the persistent activation of microglia, the innate immune cells of the CNS, with DAMPs being one of the well‐known activators of microglia [114]. In mice with spinal cord injury (SCI), high mobility group box1 (*HMGB1*) in DAMPs induces pro‐inflammatory microglia activation via the receptor for advanced glycation endproducts (RAGE)‐NF‐κB pathway, and inhibition of HMGB1 or RAGE significantly reduces neuronal loss and demyelination [115].

However, it is noteworthy that the use of the RIPK1 inhibitor Nec‐1s did not inhibit microglia‐mediated neuroinflammation, but RIPK1 displays scaffold activities in microglial necroptosis. Nonetheless, the exact mechanism of action needs to be validated by further studies [116]. Neuroinflammation and polarization of microglia (M1/M2 phenotype) and activated RIPK3/MLKL‐mediated necroptosis promote neuronal loss in an AD mouse model [117]. Moreover, RIPK3/MLKL‐mediated neuronal necroptosis regulates the M1/M2 polarization of microglia in the ischemic cortex in primary neuronal culture [118]. It was found that microglia activated by intracerebral hemorrhage (ICH) in ICH rats promote neuronal necroptosis by secreting exosomes and transferring miR‐383‐3p into neurons [119]. This suggests that necroptosis promotes neuroinflammation and neuronal loss and that targeting necroptosis promotes the conversion of M1 to M2 phenotype, restores microglial phagocytosis, and serves as a therapeutic strategy for VaD.

Astrocyte activation is also known to be closely associated with neuroinflammation. MLKL defects can reduce the loss of tyrosine hydroxylase‐positive neurons in Tg‐Mlkl mouse models and inhibit the activation of microglia and astrocytes to reduce neuroinflammation [120]. Currently, BBB disruption is the main direction of research in necroptosis of astrocytes. However, the current results indicate that inhibition of necroptosis of astrocytes can inhibit neuroinflammation, and we look forward to further studies on necroptosis of reactive astrocytes in neuroinflammation in the future.

## 4 Necroptosis Alters BBB Permeability and Exacerbates VaD

Several studies have shown that necroptosis is strongly associated with BBB permeability and that inactivation or genetic inhibition of the RIPK1 kinase reduces BBB permeability or BBB disruption, both in mouse models of ICH and experimental subarachnoid hemorrhage in rats [121, 122, 123]. There is increasing evidence that RIPK1 and RIPK3, essential kinases in necroptosis, have a role in increasing BBB permeability in cerebral ischemia or hemorrhage, and that inhibition of necroptosis may have a BBB‐maintaining and neuroprotective effect on VaD [25, 83, 122, 124, 125]. In summary, necroptosis is involved in BBB disruption. In the following sections, we will explore in detail the molecular mechanisms of BBB disruption in VaD in terms of ECs, mitochondrial dysfunction, and necroptosis involving astrocytes.

### 4.1 BBB Destruction by Necroptosis of ECs in VaD

ECs are a core component of the NVU, as they are bound together by TJs to form a selective permeability barrier between the blood and CNS, transport nutrients, and remove waste via specific receptors [126, 127, 128]. An early event in the pathogenesis of VaD is dysfunction of the ECs, leading to the loss of BBB integrity [129]. It was shown that EC‐epidermal growth factor receptor (EGFR) is a regulator of TNFR1‐mediated inflammation and RIP3‐dependent necroptosis and that inhibition of the kinase of the EGFR disrupted the formation of complex I and complex IIb and prevented RIP3‐dependent necroptosis in ECs [130]. More compelling evidence is that perivascular M1 microglia‐induced necrosis of ECs leads to BBB disruption and that anti‐TNF‐α (infliximab) treatment significantly improves ECs necroptosis, BBB disruption, and improves stroke outcomes [25]. Thus, necroptosis is a potential mechanism leading to ECs dysfunction, which ultimately leads to the destruction of the BBB, resulting in poor prognosis. Thus, preventing necroptosis of ECs in VaD is necessary to protect the BBB as well as improve the prognosis of dementia.

### 4.2 Mitochondrial Dysfunction and Oxidative Stress Promote Necroptosis and Damage the BBB

**FIGURE 3:**
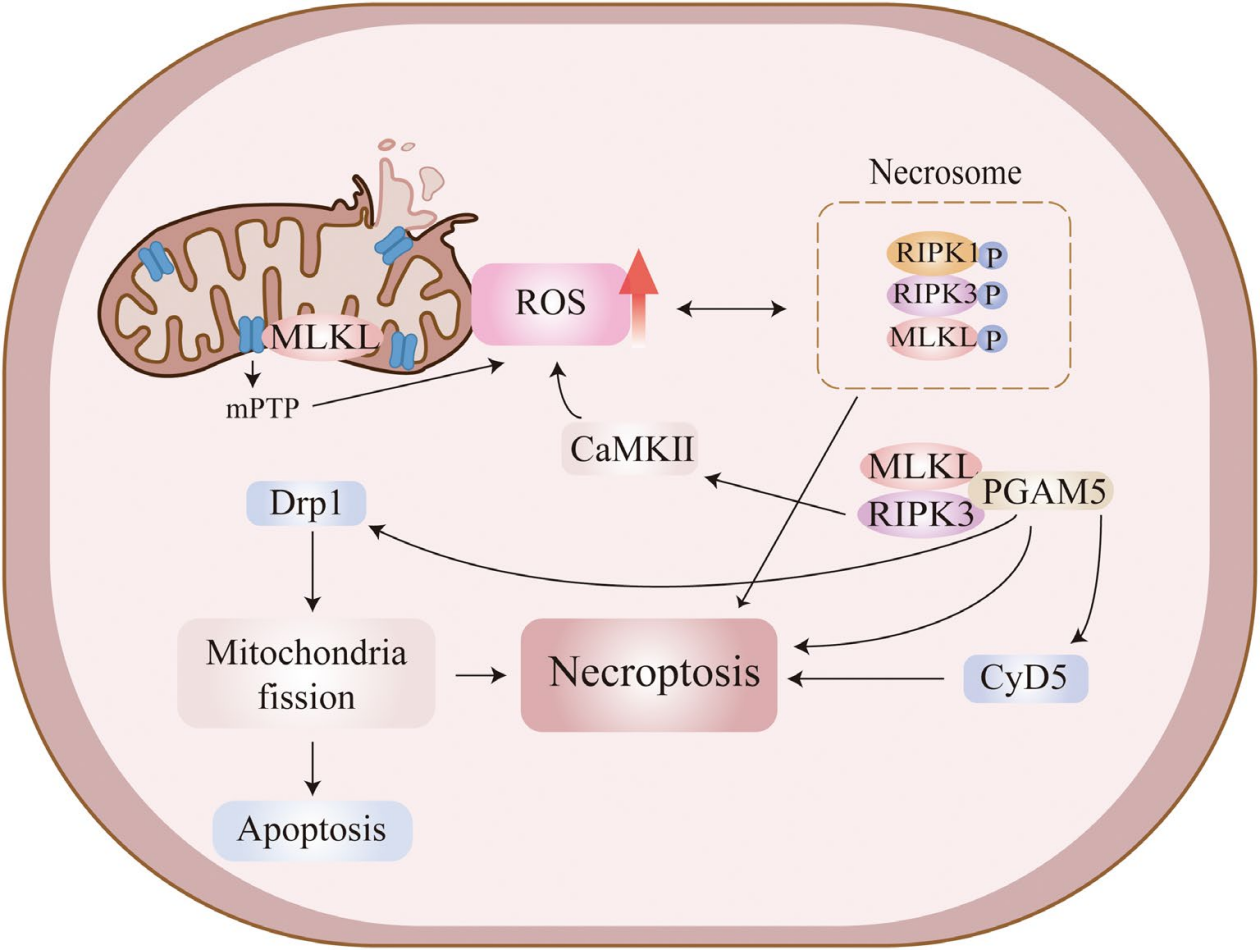
Intracellular necroptosis with mitochondrial dysfunction and signaling pathways of oxidative stress.

Mitochondrial dysfunction and oxidative stress occur in VaD; moreover, they have been shown to play a key role in the progression of VaD and the development of BBB damage [131, 132]. TNF‐α increases RIPK3 expression and promotes the formation of RIPK1/RIPK3 necrosome, leading to the production of large amounts of reactive oxygen species (ROS), causing mitochondrial dysfunction and aggravating organismal damage [133, 134]. ROS induces RIPK1 autophosphorylation on S161, which is essential for RIPK1 activation [135]. ROS activation in microglia triggers neuroinflammation and alters synaptic activity and neuronal transmission [136]. TNF‐α‐induced necroptosis requires RIPK1/RIPK3‐mediated mitochondrial ROS generation at an early stage; RIPK1 is also required to sense ROS during necroptosis execution [137]. It has been demonstrated that ROS scavenger (*N*‐Acetyl‐l‐cysteine) reduced the expression of p‐RIPK3 and p‐MLKL proteins and inhibited necroptosis after traumatic brain injury (TBI) in mice [138]. TNF‐α‐induced necroptosis requires oxidative stress‐generated ROS not only upstream but also in the downstream pathway, where phosphoglycerate mutase 5 (PGAM5), a downstream molecule of RIP3/MLKL that regulates necroptosis through activation of cyclophilin D (CypD) and dynamin‐related protein 1 (Drp1), plays an important role in oxidative stress‐induced necroptosis [139]. It has also been shown that calcium/calmodulin‐dependent protein kinase II, a downstream factor of RIPK3, can increase ROS levels and induce mitochondrial dysfunction [140]. In addition, MLKL may also be transferred to the mitochondrial membrane, leading to an over‐opening of the mitochondrial permeability transition pore (MPTP), resulting in the overproduction of ROS, which further exacerbates cell death [141] (Figure 3). In conclusion, mitochondrial dysfunction and oxidative stress play an important role in TNF‐α‐mediated necroptosis, especially for RIPK1/RIPK3 necrosome, and are responsible for the massive production of ROS, which exacerbate BBB damage and thus accelerate disease progression in VaD.

### 4.3 Effect of Astrocyte Necroptosis in the BBB on VaD

Necroptosis leads to astrocyte death, which acts as a trophic agent for neurons and maintains CNS homeostasis in vivo [142, 143, 144]. It has been demonstrated that attenuating vascular stenosis‐induced astrogliosis preserves white matter integrity and cognitive function [9]. Zhu et al. [145], in their study on ischemic stroke, showed that RIPK1 enhances the vascular endothelial growth factor D (VEGF‐D)/VEGF receptor VEGFR‐3 signaling pathways and contributes to astrogliosis and glial scar formation, and RIPK3 and MLKL are also involved in ischemia‐induced reactive astrogliosis in a transient middle cerebral artery occlusion (tMCAO) rat model and an oxygen and glucose deprivation and reoxygenation (OGD/Re)‐induced astrocytic injury model. On the other hand, the knockdown of RIPK1 prevented OGD‐induced astrocyte damage and inhibited the increase in astrocyte lysosome numbers in the ischemic cerebral cortex induced by permanent middle cerebral artery occlusion in a rat model [146]. Y‐2 inhibits astrocyte‐mediated neuroinflammation and attenuates TNF‐α‐triggered neuronal necroptosis in cell cultures and AD mice [147]. These results suggest that reducing reactive astrocyte proliferation plays an important role in the disease process of VaD. The RIPK1 inhibitor NEC‐1, which can effectively reduce astrocyte proliferation, has the potential to become an important drug to reduce the degree of dementia in VaD. However, its specific mechanism of action needs to be further investigated in depth.

M1 microglia can cause BBB dysfunction and vascular leakage, whereas M2 microglia play a protective role in the BBB [148]. Targeting necroptosis can promote the conversion of M1 to M2 phenotype. Whether it is also possible to target necroptosis in the BBB to play the protective role of M2 microglia on the BBB is something to look forward to in the future.

## 5 Treatment

### 5.1 Inhibition of Necroptosis Pathways

RIPK1 inhibitors are potent inhibitors of necroptosis [149]. Nec‐1, a blocker of RIPK1, protects the brain from ischemic necroptosis by reducing RIPK1 activation and inhibiting its downstream signaling pathways and is protective against brain injury [150, 151]. Specifically, in the middle cerebral artery occlusion (MCAO) rat model, cerebral ischemia stimulated RIPK1 phosphorylation at Ser166 residue, RIPK3 phosphorylation at Ser232 residue, and MLKL phosphorylation at Ser345 residue, and significantly increased RIPK1, RIPK3, and MLKL levels, whereas Nec‐1 attenuated these changes through inhibition of the phosphorylated RIPK1 kinase [152]. Many experiments have demonstrated that inhibition of RIPK1 activation is very effective in preventing ischemic injury [153, 154, 155]. Furthermore, in transient OGD (resupply [R]) brain microvascular ECs, administration of *Panax notoginseng* saponins and NEC‐1 could protect against OGD/R‐induced necroptosis by inhibiting phosphorylation of receptor‐interacting Ser/Thr protein kinase 1 (RIP1), RIP3, and MLKL, suggesting that *P. notoginseng* saponins and NEC‐1 effectively inhibit the occurrence of necroptosis in ischemic stroke by inhibiting the RIP1/RIP3/MLKL signaling pathways [156]. However, it is worth noting that the limited ability of anti‐TNF‐α antibodies to enter the brain makes TNF‐α antibody‐based therapies of limited utility in neurodegenerative diseases [157]. Furthermore, in necroptosis cells that have exposed phosphatidylserine in their outer membrane, targeting the pMLKL translocation was more favorable to the reversal of necroptosis events than targeting RIPK1 and RIPK3 activation [105].

The crosstalk between ferroptosis and necroptosis deserves attention. Iron overload is one of the mechanisms of ferroptosis that leads to MPTP opening, which exacerbates RIP1 phosphorylation and leads to necroptosis [158]. Furthermore, heat shock protein 90 (HSP90) induces necroptosis and ferroptosis by promoting RIP1 phosphorylation and inhibiting GPX4 activation [140]. In neuroinflammation, acyl‐CoA synthetase long‐chain family member 4 (ACSL4) overexpression exacerbates neuronal ferroptosis and pro‐inflammatory cytokine release from microglia by catalyzing the esterification of arachidonic acid and adrenergic acid to phosphatidylethanolamine [159]. *Cistanche* total glycoside capsule has been marketed to treat VaD disease. One of the active ingredients, *Cistache deserticola* phenylethanoid glycoside, can reduce the formation of lipid peroxides and improve the mechanism of ferroptosis by upregulating the GPX4/SCL7A311 axis and downregulating the ACSL4/LPCAT3/LOX axis, preventing neuroprotective effects and alleviating learning and memory dysfunctions in mice suffering from hypobaric hypoxia [160]. Thus, focusing on the association between ferroptosis and necroptosis may offer a promising therapeutic option for VaD.

### 5.2 Inhibition of Necroptosis‐Mediated Over‐Activation of Glial Cells

It has been demonstrated that fetuin‐A inhibits oxidative stress and necroptosis in glutamate‐treated primary microglia, thereby attenuating the aberrant inflammatory response after trauma [138]. Moreover, necrosulfonamide inhibits astrocyte necroptosis and improves prognosis by preventing the upregulation of protein kinase expression in necroptosis in the tMCAO rat model, blocking translocation of p‐MLKL and p‐RIPK3 to the nuclear membrane and co‐localization at the nuclear membrane [161]. It has been found that melatonin inhibits microglial necroptosis via the A20/RIP3 pathway, thereby reducing cell death, inflammation, and mitochondrial damage [162]. Inhibition of necroptosis of glial cells was demonstrated to be a key target for the treatment of abnormal glial cell death, activation, or neuroinflammation in VaD. Research in this area is still at an early stage and needs to be further strengthened. Currently, more studies in VaD are focused on targeting the TLR4/NF‐κB signaling pathway to alleviate neuroinflammation and improve cognitive impairment [163, 164]. Electroacupuncture (EA) has been recognized as a potential treatment for cognitive impairment in recent years, and it has been found that EA can inhibit neuroinflammation by reducing astrocyte necroptosis through down‐regulation of the RIP1/MLKL/TLR4 pathway in mice with SCI [165, 166].

### 5.3 Reduction of Oxidative Stress to Inhibit Necroptosis and Thus Protect Neurons

First, oxidation of vitamin C and the generation of dehydroascorbic acid could regulate neuronal necroptosis under conditions of oxidative stress in neuronal N2a and HN33.11 cell lines and cortical neurons [167]. Second, ketamine as a potent inhibitor of necroptosis may have neuroprotective effects [168]. In the VaD rat model, lycopene prevents memory deficits by inhibiting ROS levels, but whether lycopene exerts its neuroprotective effects by directly eliminating ROS or through downstream pathways activated by ROS remains unknown [169]. Novel glycosylated angiotensin‐(1–7) Mas receptor agonist PNA5 reverses cognitive deficits, reduces ROS production, and inhibited inflammatory cytokine production in a preclinical mouse model of vascular cognitive impairment and dementia (VCID) that is induced by chronic heart failure [170]. Novel materials have been developed for the treatment of VaD. Wang et al. designed a novel inhaled nanotherapeutic agent, P/D@Mn/Co_3_O_4_, which could effectively inhibit acute and chronic cerebral ischemia symptoms by scavenging ROS and damaged mitochondria from the lesion area in a rat model of stroke and VaD [171]. Neuroinflammation characterized by TNF‐α, oxidative stress characterized by ROS and superoxide dismutase were all significantly ameliorated in a rat model of permanent occlusion of the bilateral common carotid arteries (2‐vessel occlusion [2VO]) after the use of bisindole analogue (2‐(2‐(bis(5‐chloro‐1H‐indol‐3‐yl)methyl)phenoxy) aniline, compound 4ae) in a rat model of 2VO [172]. P53 is a central stress sensor in cells and is closely associated with necroptosis by regulating redox signaling [137]. Hyperbaric oxygen inhibits intracellular p53 levels in repeated cerebral ischemia–reperfusion injury rats, providing new mechanisms and laboratory evidence for the clinical treatment of VaD [173].

In summary, little work has been done to target necroptosis as a therapeutic target for VaD, and inhibition of necroptosis in specific cell types or inhibition of key factors in necroptosis appears very promising. Small molecules that inhibit necroptosis by targeting the kinase activity of RIPK1 have shown promise in several murine models of non‐infectious disease and phase‐II human clinical trials. This has triggered an investment of more than one billion dollars (USD) into the emerging class of necroptosis‐blocking drugs [174].

## 6 Conclusion

Neuroinflammation and BBB destruction play a crucial role in the disease progression of VaD. In this review, we focus on the molecular mechanisms of necroptosis action in VaD. Targeting necroptosis in VaD significantly inhibits neuroinflammation and BBB destruction and improves the prognosis of VaD, and therapeutic measures targeting necroptosis are expected to be part of the disease modification strategy in VaD. In this review, only the effects of both neuroinflammation and BBB permeability in VaD under necroptosis have been described. However, other aspects of VaD, such as demyelination and axonal degeneration have not been addressed in this study. In future studies, we will further explore the role of other aspects of necroptosis in VaD to provide a multifaceted perspective for the treatment of VaD.

## Conflicts of Interest

The authors declare no conflicts of interest.

## Data Availability

Data sharing not applicable to this article as no datasets were generated or analysed during the current study.

## References

[1] J. T. O'Brien and A. Thomas , “Vascular dementia,” Lancet 386, no. 10004 (2015): 1698–1706. 26595643 10.1016/S0140-6736(15)00463-8

[2] N. S. Rost , A. Brodtmann , M. P. Pase , et al., “Post‐Stroke Cognitive Impairment and Dementia,” Circulation Research 130, no. 8 (2022): 1252–1271. 35420911 10.1161/CIRCRESAHA.122.319951

[3] S. Selvaraji , M. Efthymios , R. S. Y. Foo , et al., “Time‐Restricted Feeding Modulates the DNA Methylation Landscape, Attenuates Hallmark Neuropathology and Cognitive Impairment in a Mouse Model of Vascular Dementia,” Theranostics 12, no. 7 (2022): 3007–3023. 35547760 10.7150/thno.71815 PMC9065201

[4] Q. Zhou , M. le , Y. Yang , et al., “Discovery of Novel Phosphodiesterase‐1 Inhibitors for Curing Vascular Dementia: Suppression of Neuroinflammation by Blocking NF‐κB Transcription Regulation and Activating cAMP/CREB Axis,” Acta Pharmaceutica Sinica B 13, no. 3 (2023): 1180–1191. 36970192 10.1016/j.apsb.2022.09.023 PMC10031254

[5] A. H. Hainsworth , H. S. Markus , and J. A. Schneider , “Cerebral Small Vessel Disease, Hypertension, and Vascular Contributions to Cognitive Impairment and Dementia,” Hypertension 81, no. 1 (2024): 75–86. 38044814 10.1161/HYPERTENSIONAHA.123.19943 PMC10734789

[6] W. M. van der Flier , I. Skoog , J. A. Schneider , et al., “Vascular Cognitive Impairment,” Nature Reviews. Disease Primers 4 (2018): 18003. 10.1038/nrdp.2018.3 29446769

[7] P. Pan , Z. Ma , Z. Zhang , et al., “Acupuncture Can Regulate the Peripheral Immune Cell Spectrum and Inflammatory Environment of the Vascular Dementia Rat, and Improve the Cognitive Dysfunction of the Rats,” Frontiers in Aging Neuroscience 13 (2021): 706834. 34349636 10.3389/fnagi.2021.706834 PMC8328226

[8] G. A. Rosenberg , N. Sullivan , and M. M. Esiri , “White Matter Damage is Associated With Matrix Metalloproteinases in Vascular Dementia,” Stroke 32, no. 5 (2001): 1162–1168. 11340226 10.1161/01.str.32.5.1162

[9] Q. Liu , M. I. H. Bhuiyan , R. Liu , et al., “Attenuating Vascular Stenosis‐Induced Astrogliosis Preserves White Matter Integrity and Cognitive Function,” Journal of Neuroinflammation 18, no. 1 (2021): 187. 34454529 10.1186/s12974-021-02234-8 PMC8403348

[10] H. H. Xiao , F. R. Zhang , S. Li , et al., “Xinshubao Tablet Rescues Cognitive Dysfunction in a Mouse Model of Vascular Dementia: Involvement of Neurogenesis and Neuroinflammation,” Biomedicine & Pharmacotherapy 172 (2024): 116219. 38310654 10.1016/j.biopha.2024.116219

[11] Y. Zhao , J. Zhang , Y. Zheng , et al., “NAD(+) Improves Cognitive Function and Reduces Neuroinflammation by Ameliorating Mitochondrial Damage and Decreasing ROS Production in Chronic Cerebral Hypoperfusion Models Through Sirt1/PGC‐1α Pathway,” Journal of Neuroinflammation 18, no. 1 (2021): 207. 34530866 10.1186/s12974-021-02250-8 PMC8444613

[12] G. L. Bowman , L. Dayon , R. Kirkland , et al., “Blood‐Brain Barrier Breakdown, Neuroinflammation, and Cognitive Decline in Older Adults,” Alzheimers Dement 14, no. 12 (2018): 1640–1650. 30120040 10.1016/j.jalz.2018.06.2857

[13] Z. Wang , T. Li , M. du , et al., “β‐Hydroxybutyrate Improves Cognitive Impairment Caused by Chronic Cerebral Hypoperfusion via Amelioration of Neuroinflammation and Blood‐Brain Barrier Damage,” Brain Research Bulletin 193 (2023): 117–130. 36577190 10.1016/j.brainresbull.2022.12.011

[14] V. Rajeev , D. Y. Fann , Q. N. Dinh , et al., “Pathophysiology of Blood Brain Barrier Dysfunction During Chronic Cerebral Hypoperfusion in Vascular Cognitive Impairment,” Theranostics 12, no. 4 (2022): 1639–1658. 35198062 10.7150/thno.68304 PMC8825579

[15] B. Shan , H. Pan , A. Najafov , and J. Yuan , “Necroptosis in Development and Diseases,” Genes & Development 32, no. 5–6 (2018): 327–340. 29593066 10.1101/gad.312561.118 PMC5900707

[16] D. Bertheloot , E. Latz , and B. S. Franklin , “Necroptosis, Pyroptosis and Apoptosis: An Intricate Game of Cell Death,” Cellular & Molecular Immunology 18, no. 5 (2021): 1106–1121. 33785842 10.1038/s41423-020-00630-3 PMC8008022

[17] R. Richard and S. Mousa , “Necroptosis in Alzheimer's Disease: Potential Therapeutic Target,” Biomedicine & Pharmacotherapy 152 (2022): 113203. 35665670 10.1016/j.biopha.2022.113203

[18] J. Zhang , L. Song , J. Jia , et al., “Knowledge Mapping of Necroptosis From 2012 to 2021: A Bibliometric Analysis,” Frontiers in Immunology 13 (2022): 917155. 35769473 10.3389/fimmu.2022.917155 PMC9234124

[19] D. R. Thal , K. Gawor , and S. Moonen , “Regulated Cell Death and Its Role in Alzheimer's Disease and Amyotrophic Lateral Sclerosis,” Acta Neuropathologica 147, no. 1 (2024): 69. 38583129 10.1007/s00401-024-02722-0

[20] M. S. Arrázola , M. Lira , F. Véliz‐Valverde , et al., “Necroptosis Inhibition Counteracts Neurodegeneration, Memory Decline, and Key Hallmarks of Aging, Promoting Brain Rejuvenation,” Aging Cell 22, no. 5 (2023): e13814. 36973898 10.1111/acel.13814 PMC10186608

[21] S. Balusu , K. Horré , N. Thrupp , et al., “MEG3 Activates Necroptosis in Human Neuron Xenografts Modeling Alzheimer's Disease,” Science 381, no. 6663 (2023): 1176–1182. 37708272 10.1126/science.abp9556 PMC7615236

[22] J. Yuan , P. Amin , and D. Ofengeim , “Necroptosis and RIPK1‐Mediated Neuroinflammation in CNS Diseases,” Nature Reviews. Neuroscience 20, no. 1 (2019): 19–33. 30467385 10.1038/s41583-018-0093-1 PMC6342007

[23] N. Tisch and C. Ruiz de Almodóvar , “Contribution of Cell Death Signaling to Blood Vessel Formation,” Cellular and Molecular Life Sciences 78, no. 7 (2021): 3247–3264. 33783563 10.1007/s00018-020-03738-x PMC8038986

[24] M. Zelic , F. Pontarelli , L. Woodworth , et al., “RIPK1 Activation Mediates Neuroinflammation and Disease Progression in Multiple Sclerosis,” Cell Reports 35, no. 6 (2021): 109112. 33979622 10.1016/j.celrep.2021.109112 PMC8917516

[25] A. Q. Chen , Z. Fang , X. L. Chen , et al., “Microglia‐Derived TNF‐α Mediates Endothelial Necroptosis Aggravating Blood Brain‐Barrier Disruption After Ischemic Stroke,” Cell Death & Disease 10, no. 7 (2019): 487. 31221990 10.1038/s41419-019-1716-9 PMC6586814

[26] Z. Yuan , S. Yi‐Yun , and Y. Hai‐Yan , “Triad3A Displays a Critical Role in Suppression of Cerebral Ischemic/Reperfusion (I/R) Injury by Regulating Necroptosis,” Biomedicine & Pharmacotherapy 128 (2020): 110045. 32460187 10.1016/j.biopha.2020.110045

[27] M. Belkhelfa , N. Beder , D. Mouhoub , et al., “The Involvement of Neuroinflammation and Necroptosis in the Hippocampus During Vascular Dementia,” Journal of Neuroimmunology 320 (2018): 48–57. 29759140 10.1016/j.jneuroim.2018.04.004

[28] W. Lv , Q. Zhang , Y. Li , et al., “Homer1 Ameliorates Ischemic Stroke by Inhibiting Necroptosis‐Induced Neuronal Damage and Neuroinflammation,” Inflammation Research 73, no. 1 (2024): 131–144. 38091015 10.1007/s00011-023-01824-x PMC10776472

[29] Y. Y. Zhang , J. J. Peng , D. Chen , et al., “Telaprevir Improves Memory and Cognition in Mice Suffering Ischemic Stroke via Targeting MALT1‐Mediated Calcium Overload and Necroptosis,” ACS Chemical Neuroscience 14, no. 17 (2023): 3113–3124. 37559405 10.1021/acschemneuro.3c00250

[30] W. T. Yan , Y. D. Yang , X. M. Hu , et al., “Do Pyroptosis, Apoptosis, and Necroptosis (PANoptosis) Exist in Cerebral Ischemia? Evidence From Cell and Rodent Studies,” Neural Regeneration Research 17, no. 8 (2022): 1761–1768. 35017436 10.4103/1673-5374.331539 PMC8820688

[31] W. Hu , X. Wu , D. Yu , et al., “Regulation of JNK Signaling Pathway and RIPK3/AIF in Necroptosis‐Mediated Global Cerebral Ischemia/Reperfusion Injury in Rats,” Experimental Neurology 331 (2020): 113374. 32502579 10.1016/j.expneurol.2020.113374

[32] M. G. Naito , D. Xu , P. Amin , et al., “Sequential Activation of Necroptosis and Apoptosis Cooperates to Mediate Vascular and Neural Pathology in Stroke,” Proceedings of the National Academy of Sciences of the United States of America 117, no. 9 (2020): 4959–4970. 32071228 10.1073/pnas.1916427117 PMC7060720

[33] P. S. Sachdev , D. Blacker , D. G. Blazer , et al., “Classifying Neurocognitive Disorders: The DSM‐5 Approach,” Nature Reviews Neurology 10, no. 11 (2014): 634–642. 25266297 10.1038/nrneurol.2014.181

[34] M. B. First , “Harmonisation of ICD‐11 and DSM‐V: Opportunities and Challenges,” British Journal of Psychiatry 195, no. 5 (2009): 382–390. 10.1192/bjp.bp.108.060822 19880924

[35] P. Sachdev , R. Kalaria , J. O'Brien , et al., “Diagnostic Criteria for Vascular Cognitive Disorders: A VASCOG Statement,” Alzheimer Disease and Associated Disorders 28, no. 3 (2014): 206–218. 24632990 10.1097/WAD.0000000000000034 PMC4139434

[36] H. C. Chui , J. I. Victoroff , D. Margolin , W. Jagust , R. Shankle , and R. Katzman , “Criteria for the Diagnosis of Ischemic Vascular Dementia Proposed by the State of California Alzheimer's Disease Diagnostic and Treatment Centers,” Neurology 42, no. 3 Pt 1 (1992): 473–480. 1549205 10.1212/wnl.42.3.473

[37] R. N. Kalaria , “Neuropathological Diagnosis of Vascular Cognitive Impairment and Vascular Dementia With Implications for Alzheimer's Disease,” Acta Neuropathologica 131, no. 5 (2016): 659–685. 27062261 10.1007/s00401-016-1571-z PMC4835512

[38] M. C. Folloso , S. G. Villaraza , L. Yi‐Wen , et al., “The AHA/ASA and DSM‐V Diagnostic Criteria for Vascular Cognitive Impairment Identify Cases With Predominant Vascular Pathology,” International Journal of Stroke 19 (2024): 17474930241252556. 10.1177/17474930241252556 PMC11408959 38651759

[39] G. Livingston , J. Huntley , A. Sommerlad , et al., “Dementia Prevention, Intervention, and Care: 2020 Report of the Lancet Commission,” Lancet 396, no. 10248 (2020): 413–446. 32738937 10.1016/S0140-6736(20)30367-6 PMC7392084

[40] L. Chouliaras and J. T. O'Brien , “The Use of Neuroimaging Techniques in the Early and Differential Diagnosis of Dementia,” Molecular Psychiatry 28, no. 10 (2023): 4084–4097. 37608222 10.1038/s41380-023-02215-8 PMC10827668

[41] S. R. Cox , D. M. Lyall , S. J. Ritchie , et al., “Associations Between Vascular Risk Factors and Brain MRI Indices in UK Biobank,” European Heart Journal 40, no. 28 (2019): 2290–2300. 30854560 10.1093/eurheartj/ehz100 PMC6642726

[42] D. M. Lipnicki , S. R. Makkar , J. D. Crawford , et al., “Determinants of Cognitive Performance and Decline in 20 Diverse Ethno‐Regional Groups: A COSMIC Collaboration Cohort Study,” PLoS Medicine 16, no. 7 (2019): e1002853. 31335910 10.1371/journal.pmed.1002853 PMC6650056

[43] B. P. H. Cho , E. L. Harshfield , M. al‐Thani , D. J. Tozer , S. Bell , and H. S. Markus , “Association of Vascular Risk Factors and Genetic Factors With Penetrance of Variants Causing Monogenic Stroke,” JAMA Neurology 79, no. 12 (2022): 1303–1311. 36300346 10.1001/jamaneurol.2022.3832 PMC9614680

[44] N. Pathan , M. K. Kharod , S. Nawab , M. di Scipio , G. Paré , and M. Chong , “Genetic Determinants of Vascular Dementia,” Canadian Journal of Cardiology 40 (2024): 1412–1423. 38579965 10.1016/j.cjca.2024.03.025

[45] Tian, Z. , X. Ji , and J. Liu , “Neuroinflammation in Vascular Cognitive Impairment and Dementia: Current Evidence, Advances, and Prospects,” International Journal of Molecular Sciences 23, no. 11 (2022): 6224. 35682903 10.3390/ijms23116224 PMC9181710

[46] S. Y. Kim , Y. J. Kim , S. Y. Cho , et al., “Efficacy of *Artemisia annua* Linné in Improving Cognitive Impairment in a Chronic Cerebral Hypoperfusion‐Induced Vascular Dementia Animal Model,” Phytomedicine 112 (2023): 154683. 36738479 10.1016/j.phymed.2023.154683

[47] A. Low , E. Mak , M. Malpetti , et al., “In Vivo Neuroinflammation and Cerebral Small Vessel Disease in Mild Cognitive Impairment and Alzheimer's Disease,” Journal of Neurology, Neurosurgery, and Psychiatry 92, no. 1 (2020): 45–52. 32917821 10.1136/jnnp-2020-323894 PMC7803899

[48] C. Cicognola , N. Mattsson‐Carlgren , D. van Westen , et al., “Associations of CSF PDGFRβ With Aging, Blood‐Brain Barrier Damage, Neuroinflammation, and Alzheimer Disease Pathologic Changes,” Neurology 101, no. 1 (2023): e30–e39. 37137722 10.1212/WNL.0000000000207358 PMC10351311

[49] C. Yang , Y. He , S. Ren , et al., “Hydrogen Attenuates Cognitive Impairment in Rat Models of Vascular Dementia by Inhibiting Oxidative Stress and NLRP3 Inflammasome Activation,” Advanced Healthcare Materials 13 (2024): e2400400. 38769944 10.1002/adhm.202400400

[50] Y. Zhang , J. Zhang , Y. Zhao , et al., “ChemR23 Activation Attenuates Cognitive Impairment in Chronic Cerebral Hypoperfusion by Inhibiting NLRP3 Inflammasome‐Induced Neuronal Pyroptosis,” Cell Death & Disease 14, no. 11 (2023): 721. 37932279 10.1038/s41419-023-06237-6 PMC10628255

[51] D. Singh , “Astrocytic and Microglial Cells as the Modulators of Neuroinflammation in Alzheimer's Disease,” Journal of Neuroinflammation 19, no. 1 (2022): 206. 35978311 10.1186/s12974-022-02565-0 PMC9382837

[52] Z. Yu , N. Jiang , W. Su , and Y. Zhuo , “Necroptosis: A Novel Pathway in Neuroinflammation,” Frontiers in Pharmacology 12 (2021): 701564. 34322024 10.3389/fphar.2021.701564 PMC8311004

[53] S. Xu , J. Lu , A. Shao , J. H. Zhang , and J. Zhang , “Glial Cells: Role of the Immune Response in Ischemic Stroke,” Frontiers in Immunology 11 (2020): 294. 32174916 10.3389/fimmu.2020.00294 PMC7055422

[54] Z. D. Wei , K. Liang , and A. K. Shetty , “Role of Microglia, Decreased Neurogenesis and Oligodendrocyte Depletion in Long COVID‐Mediated Brain Impairments,” Aging and Disease 14, no. 6 (2023): 1958–1966. 37815903 10.14336/AD.2023.10918 PMC10676788

[55] M. Sun , X. Shen , and Y. Ma , “Rehmannioside A Attenuates Cognitive Deficits in Rats With Vascular Dementia (VD) Through Suppressing Oxidative Stress, Inflammation and Apoptosis,” Biomedicine & Pharmacotherapy 120 (2019): 109492. 31593895 10.1016/j.biopha.2019.109492

[56] B. Liu , Y. Zhang , Z. Yang , et al., “ω‐3 DPA Protected Neurons From Neuroinflammation by Balancing Microglia M1/M2 Polarizations Through Inhibiting NF‐κB/MAPK p38 Signaling and Activating Neuron‐BDNF‐PI3K/AKT Pathways,” Marine Drugs 19, no. 11 (2021): 587. 34822458 10.3390/md19110587 PMC8619469

[57] N. J. Lai , E. L. Ngu , J. R. Pang , et al., “Carrageenophyte Kappaphycus Malesianus Inhibits Microglia‐Mediated Neuroinflammation via Suppression of AKT/NF‐κB and ERK Signaling Pathways,” Marine Drugs 20, no. 8 (2022): 534. 36005538 10.3390/md20080534 PMC9410251

[58] Y. Yang , X. Zhao , Z. Zhu , and L. Zhang , “Vascular Dementia: A Microglia's Perspective,” Ageing Research Reviews 81 (2022): 101734. 36113763 10.1016/j.arr.2022.101734

[59] M. Nematullah , F. Rashid , S. Nimker , and F. Khan , “Protein Phosphatase 2A Regulates Phenotypic and Metabolic Alteration of Microglia Cells in HFD‐Associated Vascular Dementia Mice via TNF‐α/Arg‐1 Axis,” Molecular Neurobiology 60, no. 7 (2023): 4049–4063. 37017907 10.1007/s12035-023-03324-9

[60] C. A. Chen , C. X. Li , Z. H. Zhang , et al., “Qinzhizhudan Formula Dampens Inflammation in Microglia Polarization of Vascular Dementia Rats by Blocking MyD88/NF‐κB Signaling Pathway: Through Integrating Network Pharmacology and Experimental Validation,” Journal of Ethnopharmacology 318, no. Pt A (2024): 116769. 37400007 10.1016/j.jep.2023.116769

[61] Y. Hase , K. E. Ameen‐Ali , R. Waller , et al., “Differential Perivascular Microglial Activation in the Deep White Matter in Vascular Dementia Developed Post‐Stroke,” Brain Pathology 32, no. 6 (2022): e13101. 35748290 10.1111/bpa.13101 PMC9616090

[62] D. Spitzer , M. I. Khel , T. Pütz , et al., “A Flow Cytometry‐Based Protocol for Syngenic Isolation of Neurovascular Unit Cells From Mouse and Human Tissues,” Nature Protocols 18, no. 5 (2023): 1510–1542. 36859615 10.1038/s41596-023-00805-y

[63] Z. Liu , Y. Tang , Z. Zhang , et al., “Engineering Neurovascular Unit and Blood‐Brain Barrier for Ischemic Stroke Modeling,” Advanced Healthcare Materials 12, no. 19 (2023): e2202638. 37075477 10.1002/adhm.202202638

[64] S. Tiedt , A. M. Buchan , M. Dichgans , I. Lizasoain , M. A. Moro , and E. H. Lo , “The Neurovascular Unit and Systemic Biology in Stroke ‐ Implications for Translation and Treatment,” Nature Reviews. Neurology 18, no. 10 (2022): 597–612. 36085420 10.1038/s41582-022-00703-z

[65] S. Schaeffer and C. Iadecola , “Revisiting the Neurovascular Unit,” Nature Neuroscience 24, no. 9 (2021): 1198–1209. 34354283 10.1038/s41593-021-00904-7 PMC9462551

[66] R. Liu , J. M. Collier , N. H. Abdul‐Rahman , O. Capuk , Z. Zhang , and G. Begum , “Dysregulation of Ion Channels and Transporters and Blood‐Brain Barrier Dysfunction in Alzheimer's Disease and Vascular Dementia,” Aging and Disease 15, no. 4 (2024): 1748–1770. 38300642 10.14336/AD.2023.1201 PMC11272208

[67] Y. Matsui , F. Muramatsu , H. Nakamura , et al., “Brain‐Derived Endothelial Cells Are Neuroprotective in a Chronic Cerebral Hypoperfusion Mouse Model,” Communications Biology 7, no. 1 (2024): 338. 38499610 10.1038/s42003-024-06030-x PMC10948829

[68] H. Zhang , J. Shang , W. Li , D. Gao , and J. Zhang , “Increased Expression of VCAM1 on Brain Endothelial Cells Drives Blood‐Brain Barrier Impairment Following Chronic Cerebral Hypoperfusion,” ACS Chemical Neuroscience 15, no. 10 (2024): 2028–2041. 38710594 10.1021/acschemneuro.4c00039 PMC11099957

[69] C. Zhou , P. Sun , Y. Xu , et al., “Genetic Deficiency of MicroRNA‐15a/16‐1 Confers Resistance to Neuropathological Damage and Cognitive Dysfunction in Experimental Vascular Cognitive Impairment and Dementia,” Advanced Science (Weinheim) 9, no. 17 (2022): e2104986. 10.1002/advs.202104986 PMC9189640 35403823

[70] E. C. Lee , D. Y. Hong , D. H. Lee , et al., “Inflammation and Rho‐Associated Protein Kinase‐Induced Brain Changes in Vascular Dementia,” Biomedicine 10, no. 2 (2022): 446. 10.3390/biomedicines10020446 PMC8962349 35203655

[71] Y. Ji , Q. Gao , Y. Ma , et al., “An MMP‐9 Exclusive Neutralizing Antibody Attenuates Blood‐Brain Barrier Breakdown in Mice With Stroke and Reduces Stroke Patient‐Derived MMP‐9 Activity,” Pharmacological Research 190 (2023): 106720. 36893823 10.1016/j.phrs.2023.106720 PMC11934118

[72] Y. L. Chai , V. Rajeev , L. Poh , et al., “Chronic Cerebral Hypoperfusion Alters the CypA‐EMMPRIN‐Gelatinase Pathway: Implications for Vascular Dementia,” Journal of Cerebral Blood Flow and Metabolism 43, no. 5 (2023): 722–735. 36537035 10.1177/0271678X221146401 PMC10108186

[73] D. A. Nation , M. D. Sweeney , A. Montagne , et al., “Blood‐Brain Barrier Breakdown Is an Early Biomarker of Human Cognitive Dysfunction,” Nature Medicine 25, no. 2 (2019): 270–276. 10.1038/s41591-018-0297-y PMC6367058 30643288

[74] Y. Wang , W. du , Y. Sun , J. Zhang , C. Ma , and X. Jin , “CRTC1 Is a Potential Target to Delay Aging‐Induced Cognitive Deficit by Protecting the Integrity of the Blood‐Brain Barrier via Inhibiting Inflammation,” Journal of Cerebral Blood Flow and Metabolism 43, no. 7 (2023): 1042–1059. 37086081 10.1177/0271678X231169133 PMC10291461

[75] D. C. Hergert , O. Gaasedelen , S. G. Ryman , J. Prestopnik , A. Caprihan , and G. A. Rosenberg , “Blood‐Brain Barrier Permeability Is Associated With Cognitive Functioning in Normal Aging and Neurodegenerative Diseases,” Journal of the American Heart Association 13 (2024): e034225. 38979810 10.1161/JAHA.124.034225 PMC11292768

[76] R. Ding , Y. Hase , K. E. Ameen‐Ali , et al., “Loss of Capillary Pericytes and the Blood‐Brain Barrier in White Matter in Poststroke and Vascular Dementias and Alzheimer's Disease,” Brain Pathology 30, no. 6 (2020): 1087–1101. 32705757 10.1111/bpa.12888 PMC8018063

[77] P. Toth , S. Tarantini , A. Csiszar , and Z. Ungvari , “Functional Vascular Contributions to Cognitive Impairment and Dementia: Mechanisms and Consequences of Cerebral Autoregulatory Dysfunction, Endothelial Impairment, and Neurovascular Uncoupling in Aging,” American Journal of Physiology. Heart and Circulatory Physiology 312, no. 1 (2017): H1–H20. 27793855 10.1152/ajpheart.00581.2016 PMC5283909

[78] X. Xia , L. Lei , S. Wang , J. Hu , and G. Zhang , “Necroptosis and Its Role in Infectious Diseases,” Apoptosis 25, no. 3–4 (2020): 169–178. 31912263 10.1007/s10495-019-01589-x

[79] T. Zhang , D. Xu , J. Liu , et al., “Prolonged Hypoxia Alleviates Prolyl Hydroxylation‐Mediated Suppression of RIPK1 to Promote Necroptosis and Inflammation,” Nature Cell Biology 25, no. 7 (2023): 950–962. 37400498 10.1038/s41556-023-01170-4 PMC10617019

[80] K. Newton , K. E. Wickliffe , D. L. Dugger , et al., “Cleavage of RIPK1 by Caspase‐8 Is Crucial for Limiting Apoptosis and Necroptosis,” Nature 574, no. 7778 (2019): 428–431. 31511692 10.1038/s41586-019-1548-x

[81] D. Xu , T. Jin , H. Zhu , et al., “TBK1 Suppresses RIPK1‐Driven Apoptosis and Inflammation During Development and in Aging,” Cell 174, no. 6 (2018): 1477–1491.e19. 30146158 10.1016/j.cell.2018.07.041 PMC6128749

[82] C. Xu , J. Wu , Y. Wu , et al., “TNF‐α‐Dependent Neuronal Necroptosis Regulated in Alzheimer's Disease by Coordination of RIPK1‐p62 Complex With Autophagic UVRAG,” Theranostics 11, no. 19 (2021): 9452–9469. 34646380 10.7150/thno.62376 PMC8490500

[83] H. Wang , W. Qi , C. Zou , et al., “NEK1‐Mediated Retromer Trafficking Promotes Blood‐Brain Barrier Integrity by Regulating Glucose Metabolism and RIPK1 Activation,” Nature Communications 12, no. 1 (2021): 4826. 10.1038/s41467-021-25157-7 PMC8355301 34376696

[84] M. Feoktistova , R. Makarov , A. S. Yazdi , and D. Panayotova‐Dimitrova , “RIPK1 and TRADD Regulate TNF‐Induced Signaling and Ripoptosome Formation,” International Journal of Molecular Sciences 22, no. 22 (2021): 12459. 34830347 10.3390/ijms222212459 PMC8617695

[85] P. Chen , Z. Zhang , J. Lei , J. Zhu , and G. Liu , “Ellagitannin Component Punicalin Ameliorates Cognitive Dysfunction, Oxidative Stress, and Neuroinflammation via the Inhibition of cGAS‐STING Signaling in the Brain of an Aging Mouse Model,” Phytotherapy Research 38 (2024): 5690–5712. 39313488 10.1002/ptr.8343

[86] Y. Tian , J. Milic , L. S. Monasor , et al., “The COP9 Signalosome Reduces Neuroinflammation and Attenuates Ischemic Neuronal Stress in Organotypic Brain Slice Culture Model,” Cellular and Molecular Life Sciences 80, no. 9 (2023): 262. 37597109 10.1007/s00018-023-04911-8 PMC10439869

[87] H. Y. Zhu , F. F. Hong , and S. L. Yang , “The Roles of Nitric Oxide Synthase/Nitric Oxide Pathway in the Pathology of Vascular Dementia and Related Therapeutic Approaches,” International Journal of Molecular Sciences 22, no. 9 (2021): 4540. 33926146 10.3390/ijms22094540 PMC8123648

[88] H. Wang , Y. Chen , X. Liu , et al., “TNF‐α Derived From Arsenite‐Induced Microglia Activation Mediated Neuronal Necroptosis,” Ecotoxicology and Environmental Safety 236 (2022): 113468. 35378400 10.1016/j.ecoenv.2022.113468

[89] X. Zhou , Y. Zhu , L. Gao , et al., “Binding of RAGE and RIPK1 Induces Cognitive Deficits in Chronic Hyperglycemia‐Derived Neuroinflammation,” CNS Neuroscience & Therapeutics 30, no. 3 (2024): e14449. 37665158 10.1111/cns.14449 PMC10916433

[90] N. Weinelt , K. N. Wächtershäuser , G. Celik , et al., “LUBAC‐Mediated M1 Ub Regulates Necroptosis by Segregating the Cellular Distribution of Active MLKL,” Cell Death & Disease 15, no. 1 (2024): 77. 38245534 10.1038/s41419-024-06447-6 PMC10799905

[91] Y. W. Nam , J. H. Shin , S. Kim , et al., “EGFR Inhibits TNF‐α‐Mediated Pathway by Phosphorylating TNFR1 at Tyrosine 360 and 401,” Cell Death and Differentiation 31, no. 10 (2024): 1318–1332. 38789573 10.1038/s41418-024-01316-3 PMC11445491

[92] J. Z. Roberts , N. Crawford , and D. B. Longley , “The Role of Ubiquitination in Apoptosis and Necroptosis,” Cell Death and Differentiation 29, no. 2 (2022): 272–284. 34912054 10.1038/s41418-021-00922-9 PMC8817035

[93] A. L. Samson , S. E. Garnish , J. M. Hildebrand , and J. M. Murphy , “Location, Location, Location: A Compartmentalized View of TNF‐Induced Necroptotic Signaling,” Science Signaling 14, no. 668 (2021): eabc6178. 33531383 10.1126/scisignal.abc6178

[94] K. Lai , J. Wang , S. Lin , et al., “Sensing of Mitochondrial DNA by ZBP1 Promotes RIPK3‐Mediated Necroptosis and Ferroptosis in Response to Diquat Poisoning,” Cell Death and Differentiation 31, no. 5 (2024): 635–650. 38493248 10.1038/s41418-024-01279-5 PMC11094118

[95] J. Li , X. Liu , Y. Liu , et al., “Saracatinib Inhibits Necroptosis and Ameliorates Psoriatic Inflammation by Targeting MLKL,” Cell Death & Disease 15, no. 2 (2024): 122. 38331847 10.1038/s41419-024-06514-y PMC10853205

[96] S. Zhou , W. Zhang , G. Cai , et al., “Myofiber Necroptosis Promotes Muscle Stem Cell Proliferation via Releasing Tenascin‐C During Regeneration,” Cell Research 30, no. 12 (2020): 1063–1077. 32839552 10.1038/s41422-020-00393-6 PMC7784988

[97] S. A. Cruz , Z. Qin , A. F. R. Stewart , and H. H. Chen , “Dabrafenib, an Inhibitor of RIP3 Kinase‐Dependent Necroptosis, Reduces Ischemic Brain Injury,” Neural Regeneration Research 13, no. 2 (2018): 252–256. 29557374 10.4103/1673-5374.226394 PMC5879896

[98] C. Custodero , A. Ciavarella , F. Panza , et al., “Role of Inflammatory Markers in the Diagnosis of Vascular Contributions to Cognitive Impairment and Dementia: A Systematic Review and Meta‐Analysis,” Geroscience 44, no. 3 (2022): 1373–1392. 35486344 10.1007/s11357-022-00556-w PMC9213626

[99] A. Murao , M. Aziz , H. Wang , M. Brenner , and P. Wang , “Release Mechanisms of Major DAMPs,” Apoptosis 26, no. 3–4 (2021): 152–162. 33713214 10.1007/s10495-021-01663-3 PMC8016797

[100] M. J. Morgan and Y. S. Kim , “Roles of RIPK3 in Necroptosis, Cell Signaling, and Disease,” Experimental & Molecular Medicine 54, no. 10 (2022): 1695–1704. 36224345 10.1038/s12276-022-00868-z PMC9636380

[101] X. Tong , R. Tang , M. Xiao , et al., “Targeting Cell Death Pathways for Cancer Therapy: Recent Developments in Necroptosis, Pyroptosis, Ferroptosis, and Cuproptosis Research,” Journal of Hematology & Oncology 15, no. 1 (2022): 174. 36482419 10.1186/s13045-022-01392-3 PMC9733270

[102] N. Kayagaki , O. S. Kornfeld , B. L. Lee , et al., “NINJ1 Mediates Plasma Membrane Rupture During Lytic Cell Death,” Nature 591, no. 7848 (2021): 131–136. 33472215 10.1038/s41586-021-03218-7

[103] W. Zhang , D. Xiao , Q. Mao , and H. Xia , “Role of Neuroinflammation in Neurodegeneration Development,” Signal Transduction and Targeted Therapy 8, no. 1 (2023): 267. 37433768 10.1038/s41392-023-01486-5 PMC10336149

[104] Y. Zhang , M. Li , X. Li , et al., “Catalytically Inactive RIP1 and RIP3 Deficiency Protect Against Acute Ischemic Stroke by Inhibiting Necroptosis and Neuroinflammation,” Cell Death & Disease 11, no. 7 (2020): 565. 32703968 10.1038/s41419-020-02770-w PMC7378260

[105] S. Zargarian , I. Shlomovitz , Z. Erlich , et al., “Phosphatidylserine Externalization, “Necroptotic Bodies” Release, and Phagocytosis During Necroptosis,” PLoS Biology 15, no. 6 (2017): e2002711. 28650960 10.1371/journal.pbio.2002711 PMC5501695

[106] B. Cappe , P. Vandenabeele , and F. B. Riquet , “A Guide to the Expanding Field of Extracellular Vesicles and Their Release in Regulated Cell Death Programs,” FEBS Journal 291, no. 10 (2024): 2068–2090. 37872002 10.1111/febs.16981

[107] Y. C. Yang , Q. Jiang , K. P. Yang , L. Wang , G. Sethi , and Z. Ma , “Extracellular Vesicle‐Mediated Ferroptosis, Pyroptosis, and Necroptosis: Potential Clinical Applications in Cancer Therapy,” Cell Death Discovery 10, no. 1 (2024): 23. 38216595 10.1038/s41420-024-01799-6 PMC10786909

[108] Z. Qiu , H. Zhang , M. Xia , et al., “Programmed Death of Microglia in Alzheimer's Disease: Autophagy, Ferroptosis, and Pyroptosis,” Journal of Prevention of Alzheimer's Disease 10, no. 1 (2023): 95–103. 10.14283/jpad.2023.3 36641613

[109] T. Bartels , S. De Schepper , and S. Hong , “Microglia Modulate Neurodegeneration in Alzheimer's and Parkinson's Diseases,” Science 370, no. 6512 (2020): 66–69. 33004513 10.1126/science.abb8587

[110] S. Sun , J. Li , S. Wang , et al., “CHIT1‐Positive Microglia Drive Motor Neuron Ageing in the Primate Spinal Cord,” Nature 624, no. 7992 (2023): 611–620. 37907096 10.1038/s41586-023-06783-1

[111] H. Wu , J. Zheng , S. Xu , et al., “Mer Regulates Microglial/Macrophage M1/M2 Polarization and Alleviates Neuroinflammation Following Traumatic Brain Injury,” Journal of Neuroinflammation 18, no. 1 (2021): 2. 33402181 10.1186/s12974-020-02041-7 PMC7787000

[112] X. Xian , L. L. Cai , Y. Li , et al., “Neuron Secrete Exosomes Containing miR‐9‐5p to Promote Polarization of M1 Microglia in Depression,” Journal of Nanobiotechnology 20, no. 1 (2022): 122. 35264203 10.1186/s12951-022-01332-w PMC8905830

[113] N. Lénárt , C. Cserép , E. Császár , B. Pósfai , and Á. Dénes , “Microglia‐Neuron‐Vascular Interactions in Ischemia,” Glia 72, no. 5 (2024): 833–856. 37964690 10.1002/glia.24487

[114] N. Thadathil , E. H. Nicklas , S. Mohammed , T. L. Lewis, Jr. , A. Richardson , and S. S. Deepa , “Necroptosis Increases With Age in the Brain and Contributes to Age‐Related Neuroinflammation,” Geroscience 43, no. 5 (2021): 2345–2361. 34515928 10.1007/s11357-021-00448-5 PMC8599532

[115] H. Fan , H. B. Tang , Z. Chen , et al., “Inhibiting HMGB1‐RAGE Axis Prevents Pro‐Inflammatory Macrophages/Microglia Polarization and Affords Neuroprotection After Spinal Cord Injury,” Journal of Neuroinflammation 17, no. 1 (2020): 295. 33036632 10.1186/s12974-020-01973-4 PMC7547440

[116] X. Li , Y. Cai , J. Luo , et al., “Metformin Attenuates Hypothalamic Inflammation via Downregulation of RIPK1‐Independent Microglial Necroptosis in Diet‐Induced Obese Mice,” Cell Death Discovery 7, no. 1 (2021): 338. 34750365 10.1038/s41420-021-00732-5 PMC8575871

[117] K. Lou , S. Liu , F. Zhang , et al., “The Effect of Hyperthyroidism on Cognitive Function, Neuroinflammation, and Necroptosis in APP/PS1 Mice,” Journal of Translational Medicine 21, no. 1 (2023): 657. 37740205 10.1186/s12967-023-04511-x PMC10517505

[118] J. Yang , Y. Zhao , L. Zhang , et al., “RIPK3/MLKL‐Mediated Neuronal Necroptosis Modulates the M1/M2 Polarization of Microglia/Macrophages in the Ischemic Cortex,” Cerebral Cortex 28, no. 7 (2018): 2622–2635. 29746630 10.1093/cercor/bhy089 PMC5998990

[119] I. Gareev , O. Beylerli , and B. Zhao , “MiRNAs as Potential Therapeutic Targets and Biomarkers for Non‐traumatic Intracerebral Hemorrhage,” Biomarker Research 12, no. 1 (2024): 17. 38308370 10.1186/s40364-024-00568-y PMC10835919

[120] L. Geng , W. Gao , H. Saiyin , et al., “MLKL Deficiency Alleviates Neuroinflammation and Motor Deficits in the α‐Synuclein Transgenic Mouse Model of Parkinson's Disease,” Molecular Neurodegeneration 18, no. 1 (2023): 94. 38041169 10.1186/s13024-023-00686-5 PMC10693130

[121] S. Lule , L. Wu , A. Sarro‐Schwartz , et al., “Cell‐Specific Activation of RIPK1 and MLKL After Intracerebral Hemorrhage in Mice,” Journal of Cerebral Blood Flow and Metabolism 41, no. 7 (2021): 1623–1633. 33210566 10.1177/0271678X20973609 PMC8221773

[122] Y. Li , C. Zou , C. Chen , et al., “Myeloid‐Derived MIF Drives RIPK1‐Mediated Cerebromicrovascular Endothelial Cell Death to Exacerbate Ischemic Brain Injury,” Proceedings of the National Academy of Sciences of the United States of America 120, no. 5 (2023): e2219091120. 36693098 10.1073/pnas.2219091120 PMC9945963

[123] S. Yuan , Z. Yu , Z. Zhang , et al., “RIP3 Participates in Early Brain Injury After Experimental Subarachnoid Hemorrhage in Rats by Inducing Necroptosis,” Neurobiology of Disease 129 (2019): 144–158. 31082470 10.1016/j.nbd.2019.05.004

[124] X. Sun , Y. Wu , F. Xu , and C. Liu , “Screening of Potent RIPK3 Inhibitors to Attenuate Necroptosis and Inflammation in Mouse Traumatic Brain Injury Models,” Experimental Neurology 372 (2024): 114633. 38061556 10.1016/j.expneurol.2023.114633

[125] R. Abdelhady , N. S. Younis , O. Ali , S. Shehata , R. H. Sayed , and R. I. Nadeem , “Cognitive Enhancing Effects of Pazopanib in D‐Galactose/Ovariectomized Alzheimer's Rat Model: Insights Into the Role of RIPK1/RIPK3/MLKL Necroptosis Signaling Pathway,” Inflammopharmacology 31, no. 5 (2023): 2719–2729. 37458952 10.1007/s10787-023-01269-y PMC10518286

[126] E. Bosseboeuf and C. Raimondi , “Signalling, Metabolic Pathways and Iron Homeostasis in Endothelial Cells in Health, Atherosclerosis and Alzheimer's Disease,” Cells 9, no. 9 (2020): 2055. Other TitleStatute TitleCommon Styles. 32911833 10.3390/cells9092055 PMC7564205

[127] G. Schiera , C. M. di Liegro , G. Schirò , G. Sorbello , and I. di Liegro , “Involvement of Astrocytes in the Formation, Maintenance, and Function of the Blood‐Brain Barrier,” Cells 13, no. 2 (2024): 150. 38247841 10.3390/cells13020150 PMC10813980

[128] E. Hochman , M. Taler , R. Flug , et al., “Serum Claudin‐5 Levels Among Patients With Unipolar and Bipolar Depression in Relation to the Pro‐Inflammatory Cytokine Tumor Necrosis Factor‐Alpha Levels,” Brain, Behavior, and Immunity 109 (2023): 162–167. 36706845 10.1016/j.bbi.2023.01.015

[129] C. Cervellati , A. Trentini , A. Pecorelli , and G. Valacchi , “Inflammation in Neurological Disorders: The Thin Boundary Between Brain and Periphery,” Antioxidants & Redox Signaling 33, no. 3 (2020): 191–210. 32143546 10.1089/ars.2020.8076

[130] H. Zhang , X. Zhang , C. Ling , et al., “EGFR‐TNFR1 Pathway in Endothelial Cell Facilitates Acute Lung Injury by NF‐κB/MAPK‐Mediated Inflammation and RIP3‐Dependent Necroptosis,” International Immunopharmacology 117 (2023): 109902. 36827922 10.1016/j.intimp.2023.109902

[131] B. Han , W. Jiang , H. Liu , et al., “Upregulation of Neuronal PGC‐1α Ameliorates Cognitive Impairment Induced by Chronic Cerebral Hypoperfusion,” Theranostics 10, no. 6 (2020): 2832–2848. 32194838 10.7150/thno.37119 PMC7052889

[132] Y. X. Chen , H. Yang , D. S. Wang , et al., “Gastrodin Alleviates Mitochondrial Dysfunction by Regulating SIRT3‐Mediated TFAM Acetylation in Vascular Dementia,” Phytomedicine 128 (2024): 155369. 38547618 10.1016/j.phymed.2024.155369

[133] Y. Zhang , S. S. Su , S. Zhao , et al., “RIP1 Autophosphorylation Is Promoted by Mitochondrial ROS and is Essential for RIP3 Recruitment Into Necrosome,” Nature Communications 8 (2017): 14329. 10.1038/ncomms14329 PMC5309790 28176780

[134] P. Wang , S. Y. Zheng , R. L. Jiang , et al., “Necroptosis Signaling and Mitochondrial Dysfunction Cross‐Talking Facilitate Cell Death Mediated by Chelerythrine in Glioma,” Free Radical Biology & Medicine 202 (2023): 76–96. 36997101 10.1016/j.freeradbiomed.2023.03.021

[135] Y. Xie , G. Zhao , X. Lei , N. Cui , and H. Wang , “Advances in the Regulatory Mechanisms of mTOR in Necroptosis,” Frontiers in Immunology 14 (2023): 1297408. 38164133 10.3389/fimmu.2023.1297408 PMC10757967

[136] Y. He , T. He , H. Li , et al., “Deciphering Mitochondrial Dysfunction: Pathophysiological Mechanisms in Vascular Cognitive Impairment,” Biomedicine & Pharmacotherapy 174 (2024): 116428. 38599056 10.1016/j.biopha.2024.116428

[137] S. Rius‐Pérez , “p53 at the Crossroad Between Mitochondrial Reactive Oxygen Species and Necroptosis,” Free Radical Biology & Medicine 207 (2023): 183–193. 37481144 10.1016/j.freeradbiomed.2023.07.022

[138] P. Zhao , Y. Wei , G. Sun , et al., “Fetuin‐A Alleviates Neuroinflammation Against Traumatic Brain Injury‐Induced Microglial Necroptosis by Regulating Nrf‐2/HO‐1 Pathway,” Journal of Neuroinflammation 19, no. 1 (2022): 269. 36333786 10.1186/s12974-022-02633-5 PMC9636801

[139] M. Cheng , N. Lin , D. Dong , J. Ma , J. Su , and L. Sun , “PGAM5: A Crucial Role in Mitochondrial Dynamics and Programmed Cell Death,” European Journal of Cell Biology 100, no. 1 (2021): 151144. 33370650 10.1016/j.ejcb.2020.151144

[140] Y. Zhou , J. Liao , Z. Mei , X. Liu , and J. Ge , “Insight Into Crosstalk Between Ferroptosis and Necroptosis: Novel Therapeutics in Ischemic Stroke,” Oxidative Medicine and Cellular Longevity 2021 (2021): 9991001. 34257829 10.1155/2021/9991001 PMC8257382

[141] M. Yang , W. Chen , L. He , D. Liu , L. Zhao , and X. Wang , “A Glimpse of Necroptosis and Diseases,” Biomedicine & Pharmacotherapy 156 (2022): 113925. 36411617 10.1016/j.biopha.2022.113925

[142] Y. Rajesh and T. D. Kanneganti , “Innate Immune Cell Death in Neuroinflammation and Alzheimer's Disease,” Cells 11, no. 12 (2022): 1885. 35741014 10.3390/cells11121885 PMC9221514

[143] Z. Wu , T. Deshpande , L. Henning , P. Bedner , G. Seifert , and C. Steinhäuser , “Cell Death of Hippocampal CA1 Astrocytes During Early Epileptogenesis,” Epilepsia 62, no. 7 (2021): 1569–1583. 33955001 10.1111/epi.16910

[144] E. V. Mitroshina , M. Saviuk , and M. V. Vedunova , “Necroptosis in CNS Diseases: Focus on Astrocytes,” Frontiers in Aging Neuroscience 14 (2022): 1016053. 36778591 10.3389/fnagi.2022.1016053 PMC9911465

[145] Y. M. Zhu , L. Lin , C. Wei , et al., “The Key Regulator of Necroptosis, RIP1 Kinase, Contributes to the Formation of Astrogliosis and Glial Scar in Ischemic Stroke,” Translational Stroke Research 12, no. 6 (2021): 991–1017. 33629276 10.1007/s12975-021-00888-3 PMC8557200

[146] H. P. Du , Y. Guo , Y. M. Zhu , et al., “RIPK1 Inhibition Contributes to Lysosomal Membrane Stabilization in Ischemic Astrocytes via a Lysosomal Hsp70.1B‐Dependent Mechanism,” Acta Pharmacologica Sinica 44, no. 8 (2023): 1549–1563. 37055533 10.1038/s41401-023-01069-8 PMC10374908

[147] C. Xu , Y. Mei , R. Yang , et al., “Edaravone Dexborneol Mitigates Pathology in Animal and Cell Culture Models of Alzheimer's Disease by Inhibiting Neuroinflammation and Neuronal Necroptosis,” Cell & Bioscience 14, no. 1 (2024): 55. 38678262 10.1186/s13578-024-01230-8 PMC11056062

[148] W. Ning , S. Lv , Q. Wang , and Y. Xu , “The Pivotal Role of Microglia in Injury and the Prognosis of Subarachnoid Hemorrhage,” Neural Regeneration Research 20, no. 7 (2025): 1829–1848. 38993136 10.4103/NRR.NRR-D-24-00241 PMC11691474

[149] Y. Bai , Y. Qiao , M. Li , et al., “RIPK1 Inhibitors: A Key to Unlocking the Potential of Necroptosis in Drug Development,” European Journal of Medicinal Chemistry 265 (2024): 116123. 38199165 10.1016/j.ejmech.2024.116123

[150] X. X. Deng , S. S. Li , and F. Y. Sun , “Necrostatin‐1 Prevents Necroptosis in Brains After Ischemic Stroke via Inhibition of RIPK1‐Mediated RIPK3/MLKL Signaling,” Aging and Disease 10, no. 4 (2019): 807–817. 31440386 10.14336/AD.2018.0728 PMC6675533

[151] A. Degterev , Z. Huang , M. Boyce , et al., “Chemical Inhibitor of Nonapoptotic Cell Death With Therapeutic Potential for Ischemic Brain Injury,” Nature Chemical Biology 1, no. 2 (2005): 112–119. 16408008 10.1038/nchembio711

[152] K. Ren , J. Pei , Y. Guo , et al., “Regulated Necrosis Pathways: A Potential Target for Ischemic Stroke,” Burns Trauma 11 (2023): tkad016. 38026442 10.1093/burnst/tkad016 PMC10656754

[153] T. Zhang , D. Xu , E. Trefts , et al., “Metabolic Orchestration of Cell Death by AMPK‐Mediated Phosphorylation of RIPK1,” Science 380, no. 6652 (2023): 1372–1380. 37384704 10.1126/science.abn1725 PMC10617018

[154] E. V. Mitroshina , M. M. Loginova , R. S. Yarkov , et al., “Inhibition of Neuronal Necroptosis Mediated by RIPK1 Provides Neuroprotective Effects on Hypoxia and Ischemia In Vitro and In Vivo,” International Journal of Molecular Sciences 23, no. 2 (2022): 735. 35054920 10.3390/ijms23020735 PMC8775468

[155] C. L. Pierotti , A. V. Jacobsen , C. Grohmann , et al., “The VEGFR/PDGFR Tyrosine Kinase Inhibitor, ABT‐869, Blocks Necroptosis by Targeting RIPK1 Kinase,” Biochemical Journal 480, no. 9 (2023): 665–684. 37115711 10.1042/BCJ20230035 PMC10212518

[156] L. Zhang , Z. Hu , Z. Li , and Y. Lin , “Crosstalk Among Mitophagy, Pyroptosis, Ferroptosis, and Necroptosis in Central Nervous System Injuries,” Neural Regeneration Research 19, no. 8 (2024): 1660–1670. 38103229 10.4103/1673-5374.389361 PMC10960298

[157] D. Lecca , Y. J. Jung , M. T. Scerba , et al., “Role of Chronic Neuroinflammation in Neuroplasticity and Cognitive Function: A Hypothesis,” Alzheimers Dement 18, no. 11 (2022): 2327–2340. 35234334 10.1002/alz.12610 PMC9437140

[158] Q. Tian , B. Qin , Y. Gu , et al., “ROS‐Mediated Necroptosis is Involved in Iron Overload‐Induced Osteoblastic Cell Death,” Oxidative Medicine and Cellular Longevity 2020 (2020): 1295382. 33123307 10.1155/2020/1295382 PMC7586162

[159] Y. Cui , Y. Zhang , X. Zhao , et al., “ACSL4 Exacerbates Ischemic Stroke by Promoting Ferroptosis‐Induced Brain Injury and Neuroinflammation,” Brain, Behavior, and Immunity 93 (2021): 312–321. 33444733 10.1016/j.bbi.2021.01.003

[160] X. Zhang , Z. Liu , Z. Li , et al., “Ferroptosis Pathways: Unveiling the Neuroprotective Power of Cistache Deserticola Phenylethanoid Glycosides,” Journal of Ethnopharmacology 333 (2024): 118465. 38944360 10.1016/j.jep.2024.118465

[161] X. Y. Zhou , B. Lin , W. Chen , et al., “The Brain Protection of MLKL Inhibitor Necrosulfonamide Against Focal Ischemia/Reperfusion Injury Associating With Blocking the Nucleus and Nuclear Envelope Translocation of MLKL and RIP3K,” Frontiers in Pharmacology 14 (2023): 1157054. 37964865 10.3389/fphar.2023.1157054 PMC10642205

[162] J. Lu , Z. Sun , Y. Fang , et al., “Melatonin Suppresses Microglial Necroptosis by Regulating Deubiquitinating Enzyme A20 After Intracerebral Hemorrhage,” Frontiers in Immunology 10 (2019): 1360. 31258534 10.3389/fimmu.2019.01360 PMC6587666

[163] J. Song , M. Li , N. Kang , et al., “Baicalein Ameliorates Cognitive Impairment of Vascular Dementia Rats via Suppressing Neuroinflammation and Regulating Intestinal Microbiota,” Brain Research Bulletin 208 (2024): 110888. 38295883 10.1016/j.brainresbull.2024.110888

[164] L. Huang , Y. Shi , and L. Zhao , “Ginkgolide B Alleviates Learning and Memory Impairment in Rats With Vascular Dementia by Reducing Neuroinflammation via Regulating NF‐κB Pathway,” Frontiers in Pharmacology 12 (2021): 676392. 34220511 10.3389/fphar.2021.676392 PMC8245850

[165] H. Zhao , X. Zong , L. Li , et al., “Electroacupuncture Inhibits Neuroinflammation Induced by Astrocytic Necroptosis Through RIP1/MLKL/TLR4 Pathway in a Mouse Model of Spinal Cord Injury,” Molecular Neurobiology 61, no. 6 (2024): 3258–3271. 37982922 10.1007/s12035-023-03650-y

[166] Y. Xin , S. Zhou , T. Chu , Y. Zhou , and A. Xu , “Protective Role of Electroacupuncture Against Cognitive Impairment in Neurological Diseases,” Current Neuropharmacology 23, no. 2 (2024): 145–171. 38379403 10.2174/1570159X22999240209102116 PMC11793074

[167] L. Ferrada , M. J. Barahona , K. Salazar , P. Vandenabeele , and F. Nualart , “Vitamin C controls neuronal necroptosis under oxidative stress,” Redox Biology 29 (2020): 101408. 31926631 10.1016/j.redox.2019.101408 PMC6938857

[168] Y. Wu , M. Zhang , H. Ke , J. Xu , H. Li , and X. Ni , “Neuroprotective Effect of Ketamine and Sevoflurane Against TNF‐α Induced Cognitive Impairment,” Environmental Toxicology 39, no. 3 (2024): 1802–1810. 38064277 10.1002/tox.24071

[169] N. W. Zhu , X. L. Yin , R. Lin , et al., “Possible Mechanisms of Lycopene Amelioration of Learning and Memory Impairment in Rats With Vascular Dementia,” Neural Regeneration Research 15, no. 2 (2020): 332–341. 31552907 10.4103/1673-5374.265565 PMC6905346

[170] C. Hoyer‐Kimura , M. Hay , J. P. Konhilas , et al., “PNA5, A Novel Mas Receptor Agonist, Improves Neurovascular and Blood‐Brain‐Barrier Function in a Mouse Model of Vascular Cognitive Impairment and Dementia,” Aging and Disease 15, no. 4 (2023): 1927–1951. 10.14336/AD.2023.0928 PMC11272189 37815905

[171] D. Wang , B. Li , S. Wang , et al., “Engineered Inhaled Nanocatalytic Therapy for Ischemic Cerebrovascular Disease by Inducing Autophagy of Abnormal Mitochondria,” NPJ Regenerative Medicine 8, no. 1 (2023): 44. 37567914 10.1038/s41536-023-00315-1 PMC10421937

[172] F. Zhang , K. Zhao , T. Tang , et al., “Bisindole Compound 4ae Ameliorated Cognitive Impairment in Rats With Vascular Dementia by Anti‐Inflammation Effect via Microglia Cells,” European Journal of Pharmacology 908 (2021): 174357. 34284012 10.1016/j.ejphar.2021.174357

[173] C. Chen , W. Chen , Z. Nong , et al., “Hyperbaric Oxygen Alleviated Cognitive Impairments in Mice Induced by Repeated Cerebral Ischemia‐Reperfusion Injury via Inhibition of Autophagy,” Life Sciences 241 (2020): 117170. 31838137 10.1016/j.lfs.2019.117170

[174] E. C. Tovey Crutchfield , S. E. Garnish , and J. M. Hildebrand , “The Role of the Key Effector of Necroptotic Cell Death, MLKL, in Mouse Models of Disease,” Biomolecules 11, no. 6 (2021): 803. 34071602 10.3390/biom11060803 PMC8227991

